# Harnessing Artificial Intelligence in Lifestyle Medicine: Opportunities, Challenges, and Future Directions

**DOI:** 10.7759/cureus.85580

**Published:** 2025-06-08

**Authors:** Diana K Saeed, Abdulqadir J Nashwan

**Affiliations:** 1 Nursing and Midwifery Research Department, Hamad Medical Corporation, Doha, QAT

**Keywords:** ai-powered virtual coaching, artificial intelligence, behavioral change interventions, chronic disease prevention, digital health, digital twin technology, lifestyle medicine, precision medicine, prediction models, wearable technology

## Abstract

Lifestyle medicine (LM) offers a transformative, evidence-based approach to preventing, managing, and potentially reversing chronic diseases by targeting modifiable lifestyle factors such as nutrition, physical activity, sleep, stress, substance use, and social connectivity. However, real-world implementation of LM is often hindered by patient adherence issues, limited clinical time, and the need for ongoing personalized support. Artificial intelligence (AI), with its capabilities in data processing, pattern recognition, and predictive modeling, presents a unique opportunity to overcome these barriers and enhance the reach and precision of LM interventions. This narrative review explores AI's integration into LM's core domains. In nutrition, AI facilitates real-time dietary assessment and personalized recommendations through image recognition and machine learning. In physical activity and fitness, AI-powered wearable devices deliver tailored feedback, support virtual coaching, and predict injury risk. AI applications in sleep medicine allow for continuous, non-invasive monitoring and the early detection of sleep disorders. AI-driven cognitive behavioral therapy chatbots and biosensor-based stress prediction tools provide scalable, cost-effective support for mental health and stress management. Moreover, AI is pivotal in chronic disease prevention by integrating lifestyle data with electronic health records to forecast disease trajectories and optimize interventions. Despite these advances, several challenges remain. Data privacy concerns, algorithmic bias, regulatory ambiguities, and varying user trust and engagement levels must be addressed to ensure equitable and ethical implementation. AI’s integration with digital twin technology and precision LM represents the next frontier in personalized health. As LM continues to evolve, AI will be indispensable in driving a more proactive, participatory, and person-centered model of care that meets the complex demands of chronic disease management in the 21st century.

## Introduction and background

Chronic noncommunicable diseases (NCDs), such as cardiovascular disease, diabetes, cancer, and respiratory conditions, are responsible for nearly three-quarters of all global deaths annually [1]. The majority of these conditions are preventable and are primarily driven by modifiable lifestyle behaviors, including poor diet, physical inactivity, chronic stress, insufficient sleep, tobacco use, and social isolation. Lifestyle medicine (LM) has emerged as a rapidly growing discipline emphasizing evidence-based behavioral interventions to prevent, treat, and often reverse chronic disease [2].

LM encompasses six core pillars: nutrition, physical activity, restorative sleep, stress management, avoidance of risky substances, and social connectivity [3]. The discipline is rooted in a holistic, person-centered model of care and focuses on empowering individuals with the tools, knowledge, and support necessary for sustainable behavior change. However, despite its proven clinical effectiveness, the real-world implementation of LM faces significant challenges. These include time constraints in clinical practice, inadequate clinician training in behavioral counseling, low patient adherence, and insufficient systems for sustained follow-up and support [4].

Against this backdrop, artificial intelligence (AI) offers a transformative opportunity to strengthen lifestyle interventions' delivery, personalization, and scalability. AI is not a monolithic technology but a suite of computational tools, including machine learning, deep learning, natural language processing, and computer vision, that can extract patterns from vast datasets, make predictions, and automate decision-making. When applied to health and wellness, AI can analyze data from wearable devices, electronic health records (EHRs), genetic profiles, and social determinants to create personalized health interventions in real time [5].

The convergence of AI and LM marks a new era of precision lifestyle medicine (PLM). This discipline adapts lifestyle interventions to population-level evidence and an individual's unique physiological, psychological, and environmental profile. Wearable biosensors can continuously monitor activity, sleep, and stress. AI algorithms can generate dynamic recommendations tailored to a user's readiness to change, genetic risk factors, and behavioral history. Virtual coaches can engage users through conversational interfaces, providing support, accountability, and real-time feedback [6].

This review explores the intersection of AI and LM through a comprehensive lens. Specifically, it aims to elucidate how AI is applied across key pillars of LM, including nutrition, physical activity, sleep, stress management, and chronic disease prevention. Additionally, it highlights AI's innovations and opportunities in enhancing patient engagement, personalization, and health outcomes. Moreover, it critically examines the ethical, social, and practical challenges accompanying AI deployment in LM. Lastly, it offers insights into future directions, including integrating AI with digital twin technology and the promise of AI-human collaboration.

## Review

The foundations of LM

LM is a clinical discipline grounded in correcting and optimizing daily habits contributing to health and disease. Unlike conventional medical models that often focus on pharmacologic or surgical interventions after disease onset, LM intervenes upstream, addressing root causes through evidence-based behavioral changes. This proactive and holistic approach prioritizes sustainable health improvements across six key domains: whole-food, plant-predominant nutrition; regular physical activity; restorative sleep; effective stress management; avoidance of risky substances (such as tobacco and excessive alcohol); and the cultivation of meaningful social connections [2].

Robust evidence underpins each pillar. Diets rich in fruits, vegetables, legumes, and whole grains are consistently associated with reductions in cardiovascular disease, type 2 diabetes, and certain cancers. Similarly, regular physical activity enhances metabolic regulation, improves mood, and reduces mortality risk. Sleep, often overlooked in traditional health promotion, plays a vital role in hormonal balance, cognitive function, and immune resilience. Chronic stress, when unmitigated, contributes to systemic inflammation and is implicated in a wide range of conditions, from depression to hypertension [7].

Despite the strength of the evidence, the real-world adoption of healthy lifestyle behaviors remains low. A multitude of barriers hinder the effective implementation of LM strategies. Individual behavior change requires motivation, self-efficacy, and consistency traits, which can undermine psychological distress, socioeconomic constraints, and competing life demands. On a systemic level, healthcare providers often face limitations in time, training, and reimbursement models that disincentivize preventive care. Furthermore, many communities - particularly those marginalized by structural inequalities - lack access to nutritious food, safe spaces for physical activity, and culturally responsive care [8].

The need for longitudinal support and individualized guidance also challenges the delivery of LM. While brief advice during clinical encounters can be helpful, meaningful and lasting change often requires personalized coaching, continuous feedback, and real-time data about the patient's environment, physiology, and habits. Traditionally, such support has required extensive human resources, making it costly and challenging to scale.

Technology, particularly AI, emerges as a powerful enabler in this context. AI-driven systems can bridge the gap between clinical intention and sustained behavior change by delivering dynamic, data-informed, and personalized lifestyle interventions at scale. Through mobile health apps, wearable sensors, virtual coaches, and predictive analytics, AI can track individual progress, identify early deviations, and offer timely, evidence-based recommendations. These tools can be incredibly impactful in enhancing engagement, accountability, and self-management, thus empowering patients to take an active role in their health journey.

AI for LM interventions

AI significantly enhances the delivery and personalization of LM by enabling continuous behavioral monitoring and analysis. One of its most potent applications lies in the real-time detection of lifestyle patterns through wearable technologies, mobile apps, and ambient sensors. These devices collect rich streams of physiological and behavioral data, including heart rate variability (HRV), sleep stages, step count, calorie expenditure, and stress biomarkers, then processed by AI algorithms to establish individual baselines and detect anomalies [9]. When deviations from these baselines are detected, such as sleep disturbance, elevated stress, or physical inactivity, systems can flag early warning signs of health deterioration and recommend tailored behavioral modifications. This level of granularity enables proactive intervention long before symptoms manifest, making AI an essential tool for preventive care within LM.

Beyond monitoring, AI is crucial in delivering dynamic and interactive lifestyle interventions. AI-powered chatbots and virtual assistants, which are grounded in cognitive behavioral therapy (CBT) frameworks, can engage users in personalized conversations that promote mental well-being, emotional resilience, and stress regulation [10]. These conversational agents are available 24/7 and provide support at moments of need, thereby addressing accessibility issues in mental health services. Simultaneously, AI-enhanced fitness and wellness platforms adjust exercise programs in real time based on individual performance, fatigue levels, and biometric data. For instance, if a user exhibits signs of overtraining or poor recovery, the AI system can recommend rest or modify the intensity of the workout to prevent injury. Such capabilities enhance safety and adherence and foster a more responsive and engaging user experience. In addition, a narrative review by Nashwan et al. highlighted the transformative role of AI in mental health, emphasizing its potential to enhance patient care through early detection, personalized interventions, remote monitoring, and ethical implementation while supporting clinical decision-making and maintaining human-centered care [11].

Furthermore, AI is propelling the evolution of precision LM, in which interventions are tailored at unprecedented specificity. By integrating traditional behavioral data with genomic information, gut microbiome profiles, and metabolomic markers, AI systems can generate wellness plans that are biologically personalized. For example, individuals with specific gene-diet interactions may receive customized nutritional guidance that aligns with their metabolic phenotype [12]. Similarly, AI can stratify users by behavioral readiness, socioeconomic background, or digital engagement level, ensuring that interventions are both biologically precise and socially and contextually relevant. This hyper-personalization positions AI as a cornerstone technology in the future of LM, bridging the gap between population-level guidelines and the nuanced needs of individual patients [13].

AI applications in the core domains of LM

AI in Nutrition and Dietary Personalization

AI has revolutionized nutritional science by enabling real-time, individualized dietary planning and assessment. Traditional methods like dietary recalls and self-reported food diaries are often hampered by user bias and inaccuracies. In contrast, AI-powered systems equipped with computer vision, deep learning, and natural language processing can analyze food images, identify portion sizes, and estimate nutrient composition with unmatched precision and speed. These tools reduce users' cognitive and reporting burden while delivering immediate and accurate feedback [14]. More broadly, AI continues to transform the nutritional landscape by enhancing personalization and improving dietary monitoring, as detailed in recent narrative reviews on its expanding role in public health and personalized nutrition [13].

In addition to enhancing monitoring, AI facilitates the development of adaptive and personalized dietary recommendations. Algorithms trained on vast datasets can now account for an individual's genetic profile, gut microbiome, food preferences, allergies, and even real-time glucose data to generate nutrition plans tailored to individual needs. For example, AI models using supervised learning have been employed in nutrigenomics to optimize macronutrient ratios based on metabolic profiles, thereby supporting better adherence and outcomes. These tools represent a move toward precision nutrition, where dietary guidance is aligned with population-level evidence and individual physiological and behavioral characteristics [15].

Moreover, AI systems continue to evolve into real-time, context-aware dietary assistants. Integrated with mobile apps and wearable devices, they can make on-the-go food recommendations, send reminders for hydration or meals, and alert users when dietary patterns deviate from set goals. In community and public health contexts, aggregated nutritional data analyzed by AI can inform policy, identify food deserts, and tailor population-level interventions. This multidimensional role of AI in nutrition contributes to a more empowered and engaged approach to dietary management in LM [16].

AI in Physical Activity and Fitness

AI is significantly enhancing the personalization and accessibility of physical fitness interventions. Wearable fitness devices with AI algorithms capture real-time data such as step count, heart rate, body temperature, respiratory rate, and even posture. These devices go beyond passive monitoring; they apply AI to interpret patterns in user activity and physiological responses, providing insights into performance, fatigue, and potential injury risks. For instance, AI can detect micro-trends that indicate overexertion or lack of recovery, prompting timely adjustments in exercise routines [5].

A particularly transformative application of AI in physical fitness is virtual coaching. AI-driven platforms like Freel tics and Apple Fitness+ deliver customized workout plans that evolve based on progress, motivation, and user feedback. These platforms use predictive analytics and reinforcement learning to fine-tune exercise duration, intensity, and variety. By doing so, they bridge the gap between professional personal training and at-home workouts, making individualized guidance accessible to a broader demographic [17].

AI also plays a critical role in preventing injuries and optimizing performance. Integrated with AI, a biomechanical analysis through motion sensors can detect gait abnormalities, posture issues, or asymmetric load distribution factors contributing to musculoskeletal injuries. Moreover, AI can help identify correlations between training load and recovery needs, promoting smarter training regimens and reducing burnout risk. These capabilities make AI a key enabler in achieving sustainable physical fitness, a vital component of LM [18].

AI in Sleep Medicine

AI has opened new frontiers in sleep medicine by translating complex biometric signals into actionable sleep health insights. Traditional sleep studies, though the gold standard, are expensive, invasive, and limited to clinical settings. In contrast, AI-powered sleep trackers utilize data from wearable devices and ambient sensors to accurately assess sleep architecture stages, latency, duration, and disturbances [19]. These tools use deep learning and signal-processing techniques to classify sleep phases and detect patterns consistent with disorders like sleep apnea, insomnia, and circadian rhythm disruptions.

Another critical advancement is longitudinal monitoring and prediction. AI enables continuous assessment of sleep quality over days or weeks, flagging early signs of sleep debt accumulation, irregular sleep-wake cycles, or stress-induced disruptions. This temporal analysis allows users and clinicians to modify habits, adjust medications, or introduce behavioral strategies before sleep issues manifest into chronic conditions. Additionally, some AI models incorporate environmental data (light, noise, and temperature) to offer contextual sleep optimization suggestions [20].

AI's role in sleep medicine extends beyond diagnostics to behavioral coaching and intervention delivery. For example, apps like Sleepio leverage neural networks to analyze user-inputted sleep diaries and provide personalized cognitive behavioral therapy for insomnia (CBT-I), guiding users through interactive modules to improve sleep hygiene, manage ruminations, and correct dysfunctional sleep beliefs [21]. Furthermore, recent clinical studies have demonstrated that digital and AI-enhanced CBT-I interventions improve neurocognitive outcomes and sleep quality, reinforcing the value of scalable, cost-effective, and tailored digital therapeutics in LM [22].

AI in Stress Management and Mental Well-Being

AI has enhanced mental health interventions in several impactful ways. Notably, AI-powered chatbots using CBT principles, such as Woebot and Wysa, can simulate therapeutic conversations, providing users with continuous support and coping strategies [10]. These interventions are cost-effective, scalable, and proven to reduce mild to moderate symptoms of depression and anxiety.

In parallel, physiological stress detection has evolved. HRV, an autonomic nervous system balance biomarker, has been widely adopted as a non-invasive stress indicator. AI models trained on HRV, galvanic skin response (GSR), and EEG data now enable accurate, real-time prediction of stress levels [9]. This supports timely interventions and allows users to understand their unique stress patterns over time.

AI in Chronic Disease Prevention and Management

AI's predictive capabilities are particularly impactful in managing lifestyle-related chronic diseases. By analyzing multimodal data such as wearable outputs, dietary intake, and historical EHRs, AI models can accurately predict disease onset and progression. In conditions like diabetes and cardiovascular disease, this facilitates early intervention and tailored care pathways [6].

**Figure 1:**
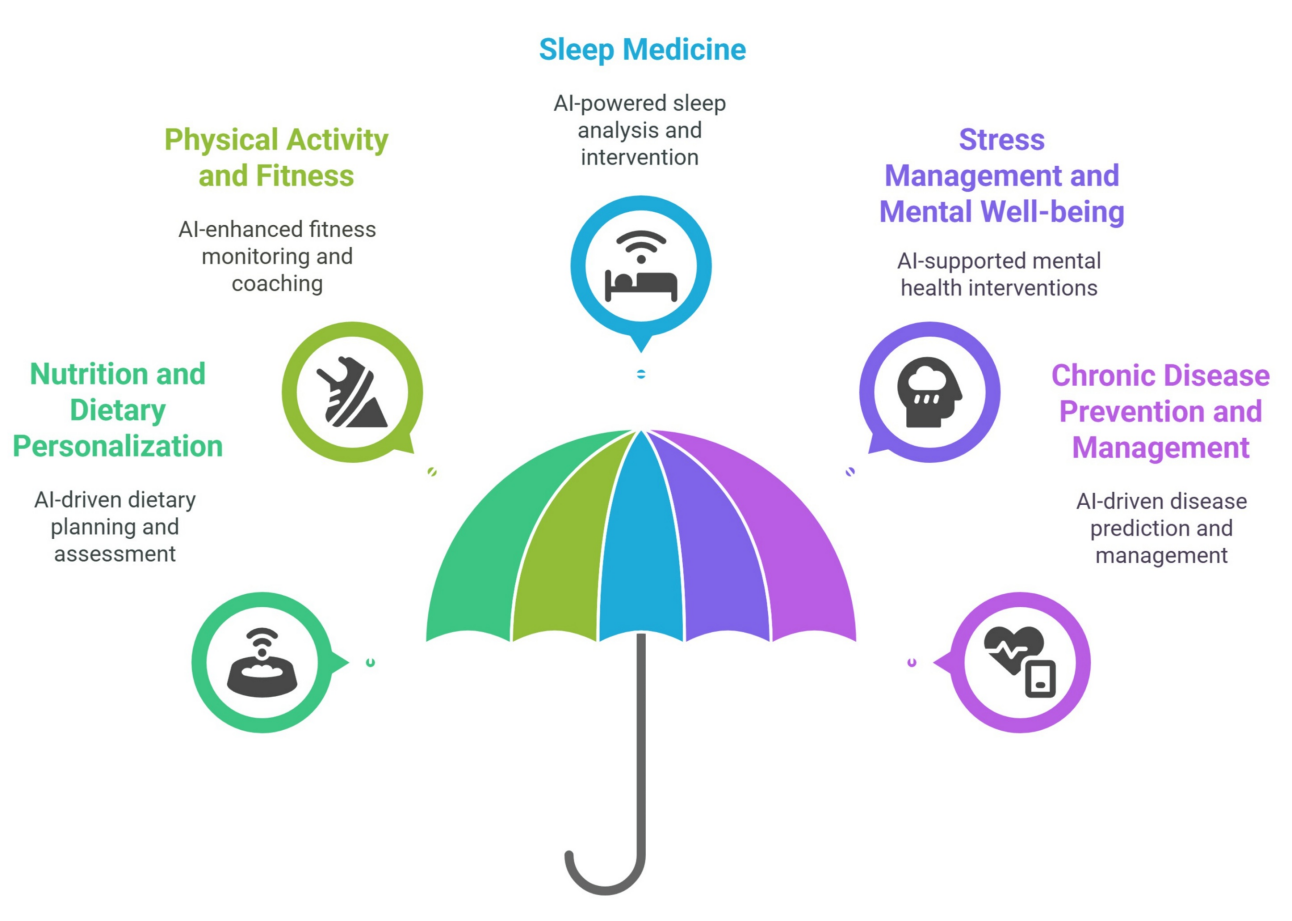
AI Applications in LM Credit: The figure was created by the author, Diana Saeed, using Napkin.ai. AI: artificial intelligence; LM: lifestyle medicine

Furthermore, AI-driven digital therapeutics integrate behavior change frameworks into mobile platforms, guiding patients in the self-management of conditions such as hypertension, obesity, and metabolic syndrome. These tools represent a shift from reactive treatment to proactive wellness promotion (Figure 1).

Ethical, social, and implementation challenges

The integration of AI into LM offers transformative potential, yet its application is entangled with complex ethical, social, and practical considerations. As AI systems increasingly participate in decision-making and behavioral guidance, the balance between innovation and responsibility becomes critical. This section explores key areas where caution, reform, and collaboration are needed to ensure equitable, trustworthy, and sustainable AI deployment in LM contexts.

Data Privacy and Security

AI systems depend on continuously ingesting and processing sensitive data, including genomic sequences, biometric readings, voice recordings, and behavioral logs. In LM, where interventions are tailored based on comprehensive lifestyle monitoring, the privacy implications are particularly profound. Many AI-powered wellness apps and digital coaching tools collect data beyond clinical settings, such as through wearables or smartphones, raising questions about informed consent, data ownership, and third-party access. While frameworks like the General Data Protection Regulation (GDPR) and the Health Insurance Portability and Accountability Act (HIPAA) offer essential protections, many consumer-grade AI tools operate in regulatory gray zones, lacking the rigorous oversight of traditional medical software [23].

Additionally, breaches of personal health data can lead to identity theft, discrimination, or stigmatization. As AI becomes more predictive, e.g., forecasting future disease risks or behavior trajectories, the consequences of such data misuse are amplified. Trust in AI-mediated LM interventions hinges on robust encryption standards, anonymization practices, transparent privacy policies, and mechanisms allowing users to control their data. Ethical AI development must also prioritize "data minimization" - collecting only what is necessary for the stated health purpose - and implement ongoing monitoring for security vulnerabilities [20].

Algorithmic Bias and Health Equity

Bias in AI systems arises when training data fails to reflect the full spectrum of human diversity. In LM, this can manifest as dietary recommendations misaligned with cultural norms. These fitness plans exclude individuals with disabilities or mental health issues that fail to detect distress in underrepresented populations. When AI models are trained primarily on data from high-income, urban, or majority-ethnic populations, their outputs may inadvertently perpetuate systemic disparities in health access and outcomes [24].

Addressing algorithmic bias requires a deliberate, proactive approach. Developers should use inclusive, representative datasets and subject models for fairness audits and performance benchmarking across demographic subgroups. Interdisciplinary collaborations with community leaders, ethicists, public health professionals, and patients can ensure that AI tools are technically accurate and socially and culturally responsive. In this way, AI can be a force not for reinforcing inequality but for advancing health equity, particularly in lifestyle-driven conditions that disproportionately affect marginalized groups.

Engagement and Trust

The success of AI in LM is not only technical but also psychological. AI tools must earn users' trust and sustained engagement to influence long-term lifestyle behavior. However, skepticism is common - patients may perceive AI recommendations as impersonal, opaque, or intrusive. There is also concern that over-reliance on AI could diminish the therapeutic alliance between clinician and patient. For these reasons, a human-AI partnership model is recommended, wherein AI supplements, not replaces, healthcare professionals' empathy, nuance, and ethical reasoning [25].

Trust-building strategies include enhancing explainability (making AI's reasoning transparent), personalizing communication styles (e.g., culturally adapted language), and incorporating user feedback into iterative design. Furthermore, clinicians must be equipped to interpret and contextualize AI outputs, translating algorithmic insights into human-centered care plans. When AI is presented as an aid to empowerment, surveillance or judgment is more likely to be embraced as a companion on the journey to lifestyle change.

Regulatory and Legal Barriers

Regulatory oversight of AI in healthcare is still catching up with its rapid development. Unlike pharmaceuticals or medical devices, many AI applications, particularly those in LM, such as virtual coaches or wellness prediction apps, are not subjected to rigorous clinical trials or post-market surveillance. This regulatory lag creates confusion around liability: if an AI system misclassifies a user's stress level or fails to flag risky behavior, who is accountable developer, the provider, or the patient? [5]

**Figure 2:**
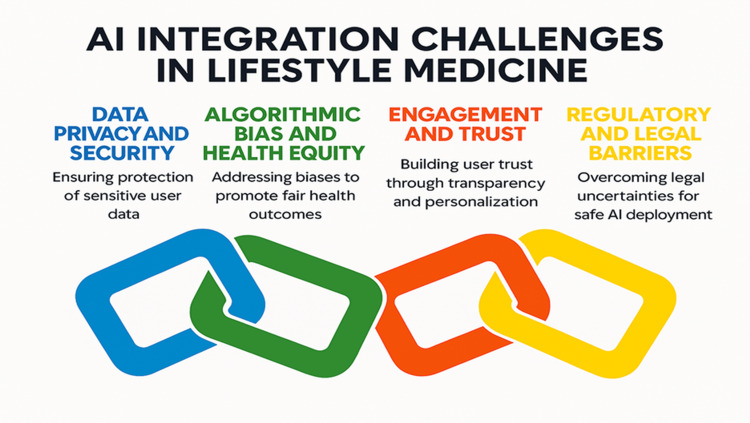
AI Integration Challenges in LM Credit: The figure was created by the author, Diana Saeed, using Napkin.ai. AI: artificial intelligence; LM: lifestyle medicine

Additionally, there is no global consensus on classifying AI tools as medical devices versus consumer wellness products, complicating approval, reimbursement, and quality assurance processes. Policymakers and regulators must work swiftly to establish frameworks encouraging innovation while safeguarding users. This includes defining standards for AI explainability, clinical validation protocols, ethical use guidelines, and mechanisms for redress. Until then, the uptake of AI in LM will remain uneven, with potential benefits constrained by unresolved legal and policy uncertainties (Figure 2).

Future directions and innovations

As AI technologies evolve, their integration into LM is expected to become more seamless, intelligent, and human-centered. The future of AI in LM lies in its ability to track and predict behavior and engage individuals, simulate outcomes, and inform systems-level strategies for sustainable well-being. The following emerging trends signal the trajectory of innovation in the field and highlight the potential of AI to advance both individual and population health outcomes.

Integration of AI With Digital Twin Technology

The digital twin technology concept, borrowed from engineering, is rapidly gaining attention in healthcare. In LM, a digital twin is a virtual replica of an individual created using real-time data from wearable sensors, EHRs, genomics, lifestyle factors, and environmental inputs. Powered by AI, this model simulates how the user's body and behavior would respond to various interventions, such as changes in diet, sleep, physical activity, or medication regimens.

These simulations offer a powerful tool for personalized lifestyle planning and risk prediction. For example, a person could test the effects of a ketogenic diet versus a Mediterranean diet or compare the impact of different stress reduction techniques before committing to one approach. Clinicians and patients can use these insights to co-create strategies more confidently, improving adherence and outcomes while reducing trial-and-error frustration.

However, deploying digital twin models at scale will require vast data interoperability, user consent mechanisms, and robust validation to avoid misinterpretation. Ethical concerns around surveillance, data misuse, and the psychological implications of viewing one's "digital future" must also be addressed. Nonetheless, this technology promises to shift the LM-moving paradigm from generalized guidelines to simulated, data-informed decision-making [26].

AI and PLM

PLM represents the convergence of AI with advances in systems biology, enabling hyper-personalized interventions. By integrating multi-omics data (genomics, metabolomics, microbiome profiles) with lifestyle patterns, AI algorithms can recommend personalized behavior changes more likely to be effective, sustainable, and aligned with an individual's unique biology.

In practice, this could mean prescribing a tailored exercise regimen based on one's mitochondrial response to activity, recommending specific foods to modulate microbiota diversity, or timing sleep interventions based on circadian genetic profiles. AI's capacity to detect subtle correlations and adapt recommendations in real time makes it the ideal engine for PLM. Moreover, as wearable technologies and at-home diagnostics improve, the cost and accessibility of these personalized insights are expected to decline.

Despite its promise, PLM must overcome challenges related to data privacy, algorithmic transparency, and access equity. Precision tools risk widening health disparities without careful regulation and design if they remain accessible only to technologically literate or affluent populations. Therefore, a commitment to inclusive design, community-based implementation, and open-access platforms will ensure that PLM fulfills its equitable potential [6].

Role of AI in Community and Public Health Initiatives

While AI is often framed in the context of individual interventions, its utility in population health management is equally significant. Through predictive analytics and pattern recognition, AI can identify high-risk populations, uncover behavioral trends, and inform policy decisions. For instance, algorithms analyzing geolocation data, food purchases, and social media can detect communities at risk of obesity, malnutrition, or mental health crises.

Public health officials can leverage these insights to deploy targeted campaigns, allocate resources more efficiently, or evaluate intervention effectiveness in real time. Moreover, AI can support behavioral nudges at scale, such as sending personalized reminders, health prompts, or incentives via SMS or app notifications. These techniques are already being tested in large-scale LM campaigns focusing on smoking cessation, physical activity promotion, and healthy eating [5].

However, using AI in public health must be balanced with ethical safeguards, especially concerning surveillance, data consent, and algorithmic fairness. Transparent partnerships between governments, academic institutions, and technology developers will be crucial to maintaining public trust. Ultimately, when integrated thoughtfully, AI can help transition LM from a niche specialty to a mainstay of public health policy and practice.

Potential for AI-Human Collaboration in Lifestyle Coaching

Rather than replacing health professionals, AI is increasingly seen as a collaborator that augments human capacity and supports shared decision-making. In LM, AI can serve as a "second brain," offering clinicians decision support by synthesizing large volumes of data and flagging emerging risks. Simultaneously, AI-powered chatbots and virtual assistants can provide continuous coaching between visits, freeing clinician time while keeping patients engaged [24].

**Figure 3:**
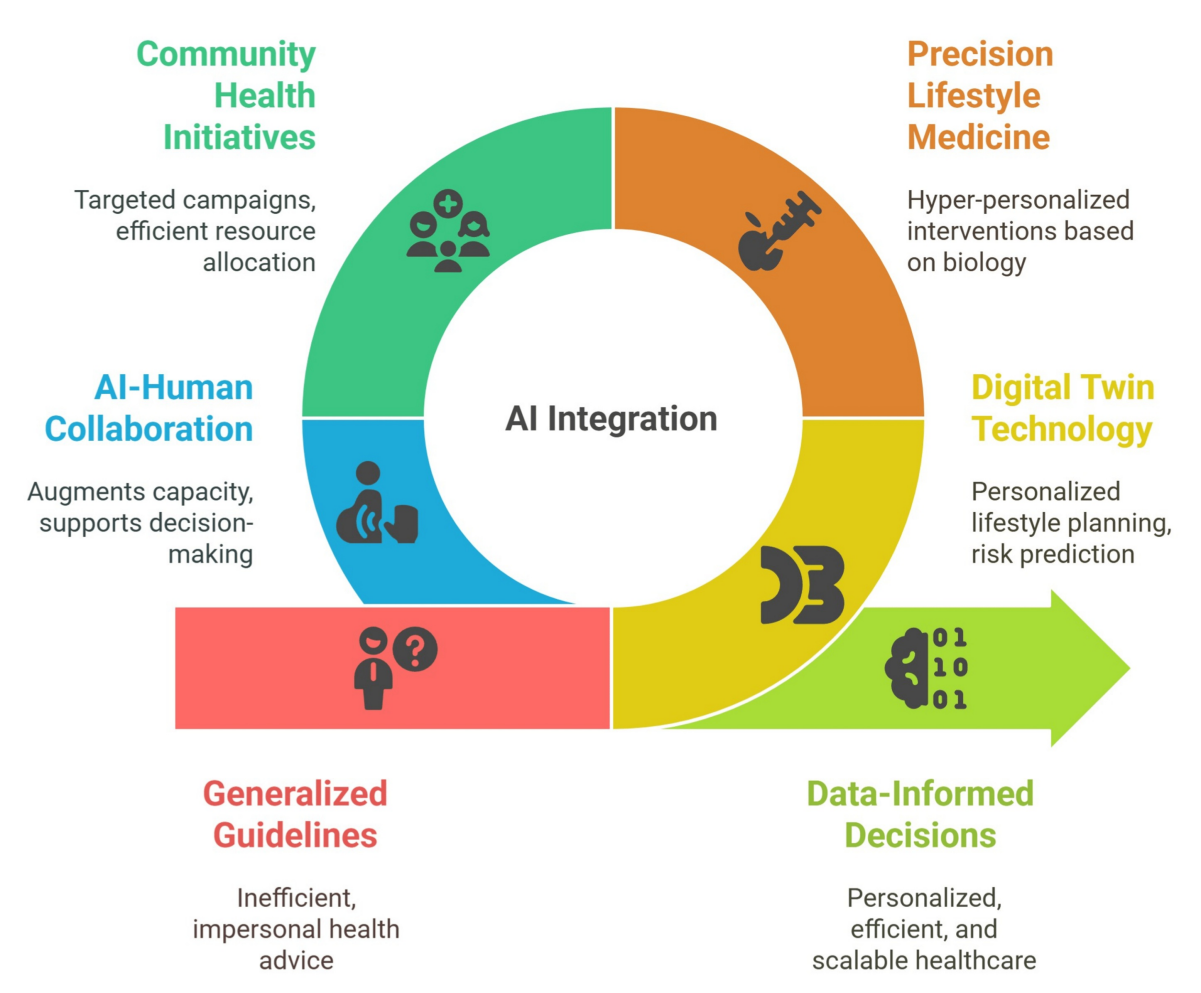
Future Directions and Innovations of AI in LM Credit: The figure was created by the author, Diana Saeed, using Napkin.ai. AI: artificial intelligence; LM: lifestyle medicine

This human-AI synergy enhances the therapeutic alliance by allowing providers to focus on empathy, motivation, and cultural context, domains in which AI still lags. For example, a clinician might rely on AI for data visualization and predictive modeling but deliver recommendations in a relational, values-based manner that aligns with the patient's goals and life circumstances. This model preserves the person-centered philosophy of the LM while improving efficiency and scale (Figure 3).

To succeed, providers must receive adequate training in digital health literacy and ethical AI use for AI-human collaboration. Patients, too, need orientation and reassurance to navigate hybrid systems confidently. AI can become a tool and a trusted partner in lifelong health behavior change through deliberate co-design and continuous feedback loops [27].

Limitations

While this narrative review comprehensively explores the intersection between AI and LM, several limitations must be acknowledged. First, the scope of the review was broad, encompassing multiple domains of LM and various AI methodologies. As a result, some specific technologies or interventions may not have been discussed in complete depth. Given the rapid pace of AI innovation, specific emerging tools and models may have been excluded due to the lag between their development and publication. Second, this review primarily synthesizes findings from peer-reviewed academic literature and grey literature sources in English, which may introduce language and publication bias. Studies published in other languages or those presented in non-traditional formats, such as conference proceedings or proprietary industry reports, were not systematically included, potentially limiting the global perspective. Third, while efforts were made to include recent and high-quality evidence, this review does not apply a formal systematic review methodology or quality appraisal tool. Therefore, the conclusions drawn are interpretative rather than quantitative and may reflect narrative biases.

Furthermore, the review does not include primary data analysis or meta-analytic techniques that would offer more substantial empirical support for its findings. Finally, the ethical, legal, and social implications of AI discussed herein evolve rapidly and may differ significantly across countries and healthcare systems. Thus, the generalizability of the identified opportunities and challenges may vary based on regional regulatory contexts, digital infrastructure, and healthcare delivery models [28].

## Conclusions

Integrating AI into LM marks a pivotal transformation in how healthcare systems can address the root causes of chronic disease. No longer limited to generic guidelines or intermittent clinician visits, LM is evolving into a dynamic, data-informed model of care, where individualized behavior change is supported by continuous monitoring, intelligent feedback, and predictive guidance. AI has proven its value across all core domains of LM, from optimizing diet and physical activity to improving sleep hygiene, managing stress, and predicting chronic disease risk. Throughout this review, we have illustrated how AI enables real-time behavior tracking, generates hyper-personalized recommendations, and scales interventions beyond the capacity of human clinicians alone. Virtual coaches, wearable sensors, and decision-support platforms are improving patient engagement and reshaping providers' roles in promoting preventive health. Yet, these technological advancements are not without limitations. Ethical considerations around privacy, equity, transparency, and regulation remain essential to ensuring that AI does not replicate or exacerbate existing healthcare disparities.

As we look to the future, the role of AI in LM will only grow more profound. Innovations such as digital twin modeling and precision LM will shift the paradigm from reactive treatment to proactive simulation, allowing individuals to test lifestyle changes before making them. Public health systems will increasingly leverage AI for predictive surveillance and behavior-informed policy design. Most importantly, the successful implementation of AI in LM will depend on fostering trust, maintaining human connection, and ensuring that technology serves as a compassionate, equitable extension of care, not a replacement for it.
